# The acute effects of dietary carbohydrate reduction on postprandial responses of non-esterified fatty acids and triglycerides: a randomized trial

**DOI:** 10.1186/s12944-018-0953-8

**Published:** 2018-12-27

**Authors:** Amirsalar Samkani, Mads J. Skytte, Christian Anholm, Arne Astrup, Carolyn F. Deacon, Jens J. Holst, Sten Madsbad, Ray Boston, Thure Krarup, Steen B. Haugaard

**Affiliations:** 1 Department of Endocrinology, Copenhagen University Hospital Bispebjerg, Bispebjerg Bakke 23, 2400 Copenhagen, NV Denmark; 2 Department of Internal Medicine, Copenhagen University Hospital, Glostrup, Denmark; 3 Department of Nutrition, Exercise and Sports, University of Copenhagen, Copenhagen, Denmark; 4 Endocrinology Research Section, Department of Biomedical Sciences, Copenhagen, Denmark; 5 Section for Translational Physiology, Center for Basic Metabolic Research, University of Copenhagen, Copenhagen, Denmark; 6 Department of Endocrinology, Copenhagen University Hospital, Amager Hvidovre, Copenhagen, Denmark; 7 School of Medicine, Department of Medicine, University of Melbourne, Melbourne, Australia; 8 Department of Internal Medicine, Copenhagen University Hospital, Amager Hvidovre, Copenhagen, Denmark

**Keywords:** Dietary carbohydrate reduction, Postprandial triglycerides, Non-esterified fatty acids

## Abstract

**Background:**

Postprandial non-esterified fatty acid (NEFA) and triglyceride (TG) responses are increased in subjects with type 2 diabetes mellitus (T2DM) and may impair insulin action and increase risk of cardiovascular disease and death. Dietary carbohydrate reduction has been suggested as non-pharmacological therapy for T2DM, but the acute effects on NEFA and TG during subsequent meals remain to be investigated.

**Methods:**

Postprandial NEFA and TG responses were assessed in subjects with T2DM by comparing a carbohydrate-reduced high-protein (CRHP) diet with a conventional diabetes (CD) diet in an open-label, randomized, cross-over study. Each diet was consumed on two consecutive days, separated by a wash-out period. The iso-caloric CRHP/CD diets contained 31/54 E% from carbohydrate, 29/16 E% energy from protein and 40/30 E% from fat, respectively. Sixteen subjects with well-controlled T2DM (median HbA_1c_ 47 mmol/mol, (37–67 mmol/mol) and BMI 30 ± 4.4 kg/m^2^) participated in the study. NEFA and TG were evaluated following breakfast and lunch.

**Results:**

NEFA net area under curve (AUC) was increased by 97 ± 38 μmol/Lx270 min (*p* = 0.024) after breakfast but reduced by 141 ± 33 μmol/Lx180 min (*p* < 0.001) after lunch on the CRHP compared with CD diet. Likewise, TG net AUC was increased by 80 ± 28 μmol/Lx270 min (*p* = 0.012) after breakfast but reduced by 320 ± 60 μmol/Lx180 min (*p* < 0.001) after lunch on the CRHP compared with CD diet.

**Conclusions:**

In well-controlled T2DM a modest reduction of dietary carbohydrate with a corresponding increase in protein and fat acutely reduced postprandial serum NEFA suppression and increased serum TG responses after a breakfast meal but had the opposite effect after a lunch meal. The mechanism behind this second-meal phenomenon of CRHP diet on important risk factors for aggravating T2DM and cardiovascular disease awaits further investigation.

**Trial registration:**

The study was registered at clinicaltrials.gov ID: NCT02472951. https://clinicaltrials.gov/ct2/show/NCT02472951. Registered June 16, 2015.

## Background

Non-esterified fatty acids (NEFAs) and triglycerides are present in the circulation in varying quantities dependent on food - and body composition, insulin resistance and glucose tolerance [1–3]. In T2DM, the adipocytes are insulin resistant, leading to a reduced anti-lipolytic effect of insulin and elevated levels of circulating NEFA especially postprandially [4]. The elevated postprandial release of NEFA has been associated with cardiovascular mortality [5] and development of type 2 diabetes mellitus (T2DM) [6, 7]*.* Enhanced postprandial triglyceridemia is a distinct component of the diabetic dyslipidemia [8] and is an independent strong predictor of ischemic heart disease [9].

In T2DM, dietary management with concomitant body weight loss is suggested to be the basis of prevention and treatment to reduce comorbidities such as cardiovascular disease [10, 11].

On the other hand, it is well-known that long-term weight loss is very difficult to obtain and another treatment strategy to improve glycemic control may be to change the quality of food recommended to patients with type 2 diabetes [12, 13]. Although the most recent consensus guideline has made no specific recommendations on the macronutrient composition [14], the Diabetes Nutrition Study Group under European Association for the Study of Diabetes stated in 2004 that the reason for recommending a moderately high carbohydrate diet was the recommendation of 15E% protein and 30E% fat, leaving 55E% for carbohydrate [15]. In diets low in carbohydrate the protein as well as the fat content is higher than in the recommended diabetes diet. The higher content of fat in carbohydrate-reduced diets could in theory induce higher postprandial concentrations of NEFA and triglycerides (TG).

The aim of the present study was to compare a carbohydrate-reduced high-protein (CRHP) and high fat diet with an iso-energetically conventional diabetes (CD) diet in relation to postprandial NEFA and TG excursions in subjects with T2DM.

## Methods

### Study design

The study design has been described in detail elsewhere [16]. In short, the participants underwent two separate 48-h interventions in a randomized, crossover design, with a 2–8 weeks washout period between each intervention. Participants were randomized to start with either a CRHP or CD diet by drawing blinded ballots, and were provided with their assigned diets, comprising breakfast, lunch, pre-dinner snack, dinner and post-dinner snack, for two consecutive days. Participants attended the Endocrine Research Unit, University of Copenhagen, Bispebjerg Hospital, Denmark, where mixed meal tests (MMT) were performed at breakfast and lunch on both days for each diet. After an overnight fast and voiding the bladder, patients were weighed, and a venous catheter was placed in an antecubital vein to draw blood samples at times − 10, 0, 10, 20, 30, 45, 60, 90, 120, 150, 180, 210, 240, 270, 280, 290, 300, 315, 330, 360, 390, 420 and 450 min (min). The breakfast provided 30% of daily total energy expenditure (TEE) and was served at time 0 min and consumed within 30 min. Likewise the lunch meal comprised 30% of TEE and was served at time 270 min and consumed within 30 min. The participants rested in a reclined position and remained sedentary throughout breakfast and lunch MMT. Dinner (30% of TEE) and snacks (10% of TEE) were provided for later consumption at home. TEE was estimated based on dual-energy X-ray absorptiometry (Lunar iDXA, GE Healthcare) prior to interventions, as described in detail elsewhere [16]. All ingredients were weighed out by the hospital research kitchen. A standardized isocaloric dinner (macronutrient content: 54% of energy from carbohydrates, 16% from protein and 30% from fat) was provided for the evening meal prior to each 48-h intervention period. Participants were asked to refrain from alcohol and strenuous physical activity for 3 days prior to each experimental period, and coffee or tea was not allowed during the intervention days.

### Participants

Sixteen participants with well-controlled T2DM in metformin mono-therapy were included in the study (Table 1). All participants were non-smokers and were weight stable throughout the study. Ten participants were treated with statins. Lipid lowering agents were kept unchanged throughout the study. Diagnosis of T2DM was based on the American Diabetes Association criteria [17].

**Table 1:** Baseline characteristics of participants, *n* = 16

Characteristics	Values *(mean ± SD)*
*N*	16
Gender (male / female)	14 / 2
Lipid lowering medication (statins / no therapy)	10 / 6
Age (y)	62 ± 6.9
Diabetes duration (y)	6.6 ± 4.6
Body weight (kg)	94 ± 17
BMI (kg/m^2^)	30 ± 4.4
HbA1c (mmol/mol)	50 ± 9.2
Fasting plasma glucose (mmol/L)	8.2 ± 2.0
Fat mass (kg)	32 ± 9.2
Fat free mass (kg)	62 ± 10
TEE^*1*^ (MJ/day)	10 ± 1.4
HOMA2-IR^2^	2.4 ± 1.0

^1^Total energy expenditure

^2^HOMA2 Calculator Version 2.2.3, University of Oxford

Written informed consent was obtained from all participants prior to any study related procedures. The study protocol was approved by the Health Ethics Committee of Copenhagen in accordance with the Helsinki-II declaration, and the trial was registered at clinicaltrials.gov (ID: NCT02472951). No participants dropped out of the study.

### Diet compositions

A CRHP diet (macronutrient composition: 31% of energy from carbohydrates, 29% from protein and 40% from fat) was compared with a CD diet (macronutrient composition: 54% of energy from carbohydrates, 16% from protein and 30% from fat) composed in accordance with the recommended diet for T2DM [15]. Full details on ingredients in the diets have been published elsewhere [16].

### Analytical procedures

The first 2 mL of each blood sample drawn were discarded, after which blood was collected in clot activator tubes that were left for 30 min before centrifugation to obtain serum. Separate samples, taken into smaller EDTA-containing tubes were centrifuged immediately for measurement of plasma glucose (PG) using YSI 2300 STAT plus (Yellow Spring Instruments, Yellow Springs, OH, USA). Serum NEFA was analyzed using a commercially available reagent (Wako, NEFA-HR [2], Wako Chemicals GmbH, Neuss, Germany) with the ACS-ACOD Method [18, 19], providing CV < 1.5% at all NEFA concentrations. Serum TG was analyzed by enzymatic colorimetric analysis by using the Cobas 8000 modular analyzer (Roche Diagnostics, Indianapolis, IN, USA) standardized against isotope dilution-mass spectrometry, providing CV of 1.4% at 2.12 mmol/L. Cortisol was measured in plasma, obtained in EDTA-tubes centrifuged at 4 degrees Celsius after sampling, by use of the Immulite 2000 Cortisol Systems Analyzer (Siemens Healthcare).

### Calculations and statistics

Area under curve (AUC) was calculated using the trapezoidal rule and net AUC was calculated by subtracting the AUC below baseline values. Values are expressed as means ± SEM. Fasting concentrations (baseline) were calculated as means of the − 10 and 0 min samples for breakfast and baseline level as the 270-min sample for lunch (initiation of lunch). To evaluate statistical differences between diets, means of both consecutive days on each diet were compared using Student’s *t* tests or Wilcoxon signed-rank test as appropriate. Graphical evaluation was used to evaluate Gaussian distribution. To evaluate differences in serum NEFA and TG concentrations at individual time points, two-way repeated measures ANOVA was used on means of both consecutive days, with subjects as fixed effects and treatment and time as repeated measures. Bonferroni’s multiple comparison adjustment of significance levels was made on individual time points as post hoc comparisons to adjust for multiple comparisons. Correlation was evaluated with Pearson or Spearman correlation as appropriate. Significance level was set at *p*-values below 0.05. With *n* = 16 participants an estimated effect size between diets of 11% (SD = 15%) was calculated to be found, provided a power of 80% at significance *p* < 0.05. GraphPad Prism (version 7.02; Graphpad Software, CA, USA) was used for statistical analyses.

## Results

Glucose and insulin data have been previously published [16]. Essentially, postprandial glucose and insulin AUC were reduced by 14 and 22% (both *p* < 0.001), respectively, by the CRHP compared with the CD diet. Secondary analyses on previously published insulin data were performed for the present study with permission from British Journal of Nutrition.

### NEFA

**Fig. 1:**
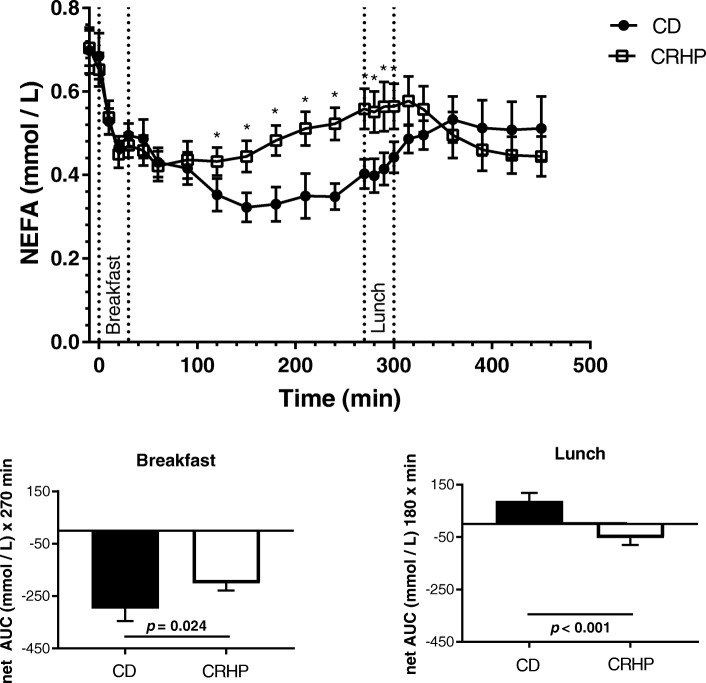
Concentration of non-esterified fatty acid (NEFA) in sixteen well-controlled patients with type 2 diabetes after intake of a carbohydrate-reduced high-protein (CRHP) or conventional diabetes (CD) breakfast (0–270 min) and lunch (270–450 min), respectively (means of 2 consecutive days on each diet). Values are presented as means with their standard errors. Bar charts show net area under curve for breakfast and lunch, respectively. *Significant difference (*p* < 0.05) between diets

Fasting NEFA concentrations did not differ on the two diets (Fig. 1). During breakfast, suppression of NEFA was more pronounced on CD resulting in a less total AUC of NEFA compared with CRHP diet (Fig. 1, Table 2). Nadir NEFA concentration was increased by 26% (354 ± 34.4 vs. 282 ± 31.7 μmol/L, *p* = 0.012) and time to nadir was decreased (114 ± 19.4 vs. 163 ± 11.0 min, *p* = 0.041), respectively, by the CRHP compared with CD diet (Fig. 1, Table 2). Net NEFA AUC was significantly greater with CRHP (− 201 ± 38 vs. − 298 ± 48 μmol/L × 270 min (*p* = 0.024)) compared with CD diet.

**Table 2:** Postprandial NEFA, triglyceride and insulin and fasting cortisol in subjects with type 2 diabetes, *N* = 16

	CD diet		CRHP diet		*p*-value^*^
	mean	SEM	mean	SEM	
NEFA responses					
Total AUC (μmol/L × 450 min)	432	35	488	34	0.003
Breakfast AUC (μmol/L × 270 min)	393	33	477	32	< 0.001
Breakfast net AUC (μmol/L × 270 min)	-298	48	-201	38	0.024
Lunch AUC (μmol/L × 180 min)	491	49	505	48	0.52
Lunch net AUC (μmol/L × 180 min)	88	31	−53	27	< 0.001
Triglyceride responses					
Total AUC (mmol/L × 450 min)	2.65	0.29	2.35	0.17	0.12
Breakfast AUC (mmol/L × 270 min)	2.43	0.26	2.21	0.16	0.20
Breakfast net AUC (mmol/L × 270 min)	0.36	0.05	0.44	0.04	0.012
Lunch AUC (mmol/L × 180 min)	2.97	0.33	2.57	0.21	0.065
Lunch net AUC (mmol/L × 180 min)	0.24	0.06	−0.08	0.05	< 0.001
Insulin responses					
Total AUC (pmol/L × 450 min)	342	36	266	31	< 0.001
Breakfast AUC (pmol/L × 270 min)	325	37	259	31	< 0.001
Breakfast net AUC (pmol/L × 270 min)	224	23	153	17	< 0.001
Lunch AUC (pmol/L × 180 min)	367	36	278	34	0.005
Lunch net AUC (pmol/L × 180 min)	171	23	80	18	0.003
Cortisol					
Fasting concentration (nmol/L)	380	26	371	34	0.71

*Abbreviations: NEFA, non-esterified fatty acids; CD, conventional diabetes; CRHP carbohydrate-reduced high-protein; AUC, area under curve;* ^*^ *using Student’s paired t-test*

Before start of lunch (270 min) baseline NEFA concentration was significantly higher after CRHP compared with CD diet (Fig. 1). The pattern of NEFA concentrations during lunch differ between the two diets with a significant increase in NEFA during CD, while a significant reduction in NEFA was observed during CRHP diet. Total AUC of NEFA during the lunch did not differ, while net AUC from baseline (88 ± 31 vs. -53 ± 27 μmol/ × 180 min, *p* < 0.001) was significantly less after CRHP compared with CD diet (Fig. 1, Table 2). At end of lunch the NEFA concentrations did not differ between the two diets.

During both meals total AUC NEFA was significantly enhanced by 13% (56 ± 16 μmol/L × 450 min, *p* = 0.003) with CRHP compared with CD (Table 2).

As a measure of within-subject variability, a strong correlation was found between fasting NEFA concentrations on each diet (Pearson *r* = 0.80, *p* < 0.001). No correlation was found between NEFA concentration at 270 min (initiation of lunch) and the increase of glucose [16] after ingestion of lunch on either diet (Pearson *r* = − 0.126 CD diet and *r* = − 0.180 CRHP diet, both *p* > 0.5), data not shown. A borderline significant correlation was found between fasting cortisol and fasting NEFA concentrations (Pearson *r* = 0.245, *p* = 0.051).

*Triglyceride.*

**Fig. 2:**
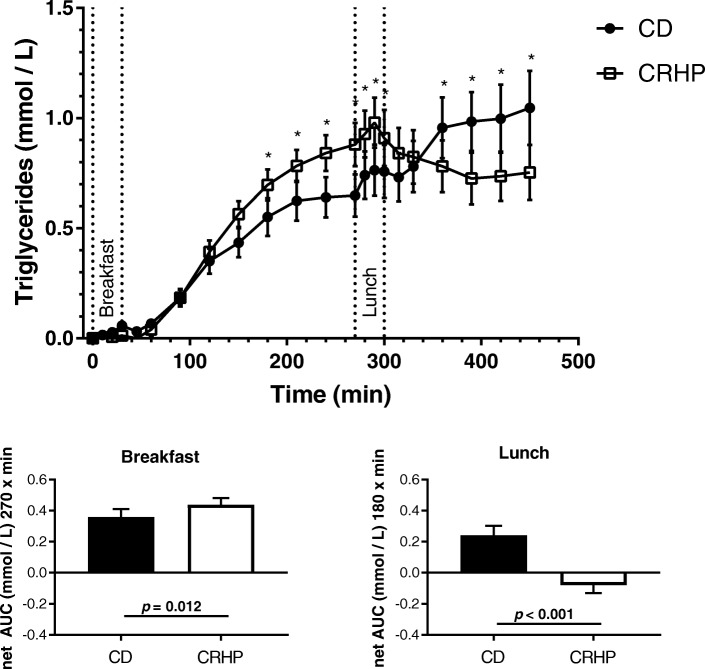
Incremental concentration of triglyceride in sixteen well-controlled patients with type 2 diabetes after intake of a carbohydrate-reduced high-protein (CRHP) or conventional diabetes (CD) breakfast (0–270 min) and lunch (270–450 min), respectively (means of 2 consecutive days on each diet). Values are presented as means with their standard errors. Bar charts show net area under curve for breakfast and lunch, respectively. *Significant difference (*p* < 0.05) between diets

Fasting TG on the CD vs. CRHP diet did not differ (1.86 (IQR 1.55;2.44) vs. 1.72 (IQR 1.40;2.07), *P* > 0.05). Peak serum TG concentrations were reached 270 min after breakfast on both diets. The increase in serum TG was enhanced after ingestion of CRHP compared with CD diet, resulting in a tendency to a higher total AUC TG and a significantly greater net AUC TG 0.44 ± 0.04 vs. 0.36 ± 0.05 mmol/L × 270 min (*p* = 0.012), respectively (Fig. 2, Table 2). Incremental TG values are presented in Fig. 2. At 270 min the TG concentration was significantly higher during CRHP compared with CD diet (Fig. 2).

After Lunch the changes in TG concentrations parallel the changes in NEFA with an increase during CD and a significant reduction in TG after CRHP diet (Fig. 2), resulting in a net AUC of − 0.08 ± 0.05 vs. 0.24 ± 0.06 mmol/L × 180 min (*p* < 0.001), respectively (Fig. 2). Lunch total AUC TG tended to be high at CD compared with CRHP diet (Table 2, *p* = 0.065).

During both meals total AUC TG did not differ significantly (Table 2, *p* = 0.12).

As a measure of within-subject variability, a strong correlation was found between fasting TG concentrations on each diet (Spearman *r* = 0.82, *p* < 0.001).

### Insulin

**Fig. 3:**
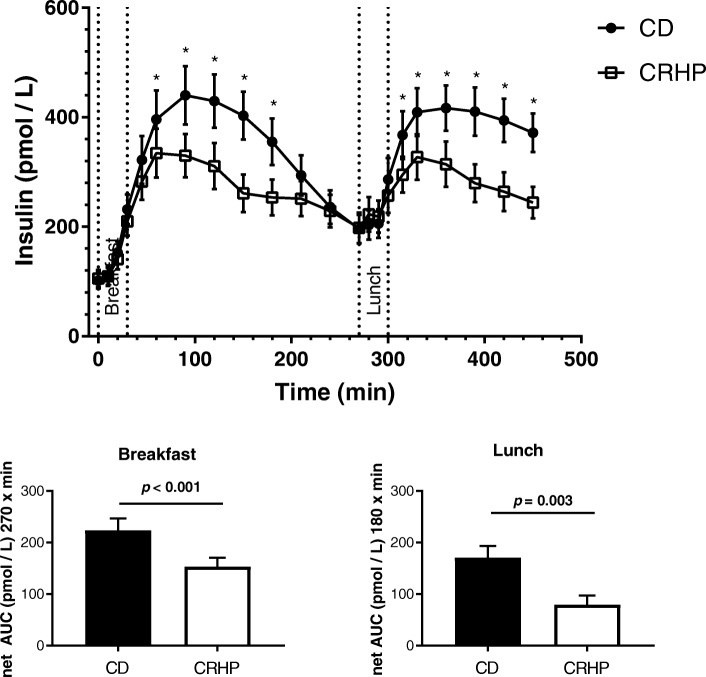
Concentration of insulin in sixteen well-controlled patients with type 2 diabetes after intake of a carbohydrate-reduced high-protein (CRHP) or conventional diabetes (CD) breakfast (0–270 min) and lunch (270–450 min), respectively (means of 2 consecutive days on each diet). Values are presented as means with their standard errors. Bar charts show net area under curve for breakfast and lunch, respectively. *Significant difference (*p* < 0.05) between diets. (Reproduced from Samkani et al., BJN, 2018, with approval from British Journal of Nutrition)

Fasting insulin concentrations did not differ between the two diets (Fig. 3). The CRHP diet reduced postprandial insulin responses after both the breakfast and lunch meals as compared with the CD diet (Fig. 3, Table 2), as peak insulin concentrations were reduced following both breakfast (334 ± 44.5 vs. 440 ± 53.6 pmol/L, *p* = 0.032) and lunch (372 ± 41.9 vs. 417 ± 41.2, *p* = 0.043) and net insulin AUC was reduced by 32% (153 ± 17 vs. 224 ± 23 pmol/L × 270 min, *p* < 0.001) following breakfast and by 53% (80 ± 18 vs. 171 ± 23 pmol/L × 180 min, *p* = 0.003) following lunch. Postprandial excursions of insulin were similar following breakfast and lunch within each diet, as no differences were found between the insulin peaks on the CRHP breakfast compared with CRHP lunch (*p* = 0.42) or on the CD breakfast compared with CD lunch (*p* = 0.82).

A correlation was found between fasting insulin and fasting TG concentrations (Pearson *r* = 0.311, *p* = 0.013), while no correlation was found between fasting insulin and fasting NEFA concentrations (Pearson *r* = 0.065, *p* = 0.61).

## Discussion

The present study showed, in well-controlled T2DM, that a modest reduction of dietary carbohydrate content with a corresponding increase in protein and fat content compared with an energy-matched conventionally diabetes diet acutely reduced postprandial serum NEFA suppression and increased serum TG responses after a breakfast meal but had the opposite effect after a lunch meal. We previously reported in the same subjects that the reduced carbohydrate intake exerted a marked acute beneficial effect on glucose excursions despite reduced insulin secretion [16].

Elevated fasting concentration of NEFA has been shown to be proportional to body fat storage [20], and reflects insulin resistance in adipose tissue and is a predictor of subsequent development of T2DM [6, 21].

In the fasting state, NEFA is released to the circulation by lipolysis in adipose tissue, primarily from subcutaneous adipose tissue, but as obesity progresses, visceral adipose tissue contributes relatively more to the NEFA pool [22]. The circulating NEFA pool provides energy substrates for tissues, with fasting circulating NEFA concentrations being regulated by hormones with diurnal variations such as cortisol, catecholamines and insulin [23–25]. In the present study, a borderline significant correlation was found between fasting cortisol and fasting NEFA concentrations.

In the fed state, insulin suppresses lipolysis and increases re-esterification of fatty acids [26, 27]. In the present study, circulating NEFA concentrations were suppressed to a smaller extent by the CRHP breakfast than by the CD breakfast, likely to be explained by the observed reduction in the postprandial insulin excursion following the CRHP diet (Fig. 3, Table 2).

An inverse response in NEFA concentrations was found after lunch, despite similar insulin concentrations following breakfast and lunch following each diet. Thus, a reduction of net NEFA AUC was found following the CRHP lunch compared with a net increase following the CD lunch. This paradoxical shift may be explained by the excursions of TG and a possible increase in lipo-protein lipase (LPL) activity facilitated by the increase in insulin [23]. Lipo-protein lipase is induced by insulin after a delay and hydrolyses TG from lipoproteins at the capillary endothelial luminal surface, where some, but not all, of the NEFA is taken up by the tissue. A fraction of of this NEFA escapes uptake and is spilled over back to the circulating NEFA pool [28–31]. We previously reported this paradoxical difference in NEFA response to breakfast and lunch in non-diabetic subjects [32]. As elevated serum NEFA concentrations can impair peripheral glucose uptake by inhibition of insulin-stimulated glucose disposal [33–35], an enhanced or reduced postprandial NEFA response may modulate postprandial glucose responses [34].

The TG excursion was increased following the CRHP breakfast, but reduced following lunch, compared with the CD diet. Thus, the second meal elicited the opposite effect on TG concentrations after the CRHP lunch compared with breakfast even though the fat content was higher in the CRHP diet. Again, the decrease in postprandial insulin response following the CRHP, compared with CD, diet (Fig. 3, Table 2) adds to the explanation of this variation. Chen et al. found a high-carbohydrate diet (55E%) to increase production of very low density lipo-protein triglyceride (VLDL-TG) in the liver of subjects with type 2 diabetes, secondary to a higher insulin concentration [36]. Both de novo lipogenesis and fatty acid re-esterification contribute to the production of VLDL-TG [37]. Chylomicron-derived TG is cleared competitively through the same pathways as VLDL-TG produced in the liver, thus explaining the net result of elevated plasma TG on higher, compared with lower, carbohydrate diets found in previous studies [36, 38–40]. This finding is only apparent in the subsequent meal after breakfast in the present study, which underscores the need to employ consecutive meals to evaluate dietary effects on lipid metabolism. Thus, interpretation of NEFA and TG excursions following a single meal may be confounded by the delayed effects of insulin on lipid metabolism. Furthermore, postprandial NEFA excursions should be evaluated in concert with TG excursions, as the circulating concentrations of these substrates are highly interdependent.

Within-subject variability of fasting and postprandial NEFA concentrations is substantial (CV 24–45%) [41, 42], and even short term (30 days) within-subject concentrations of two fasting NEFA measurements only show a correlation coefficient (r) of 0.70 (*p* < 0.001) [43], complicating use and interpretation of fasting NEFA levels. Therefore, it is crucial to employ rigorous protocols to assure standardization between interventions, e.g. equal durations of overnight fast, identical dinners prior to intervention days etc.

A strength of the present study was the thoroughly standardized protocol, designed to minimize within-subject variability between interventions, resulting in a stronger within-subject correlation (Pearson *r* = 0.80, *p* < 0.001) in fasting NEFA concentrations than previously reported. A further strength is the measurement of postprandial NEFA and TG responses to two subsequent meals, because, as was found, the response to a second meal can differ from the response to the first meal. Importantly, all meals were provided to the patients and the two diets were energy-matched to ensure a highly controlled setting.

## Conclusions

In well-controlled T2DM patients, intake of an experimental diet moderately reduced in carbohydrate and increased in protein and fat content acutely reduced postprandial serum NEFA suppression and increased serum TG responses after a breakfast meal but had the opposite effect after a lunch meal when compared with the recommended diet for diabetes patients. Studies are needed to evaluate these effects in longer term studies and in subjects with more dysregulated diabetes.

## Abbreviations

AUC: area under curve
CD: conventional diabetes
CRHP: carbohydrate-reduced high-protein
LPL: lipoprotein lipase
MMT: mixed meal test
NEFA: non-esterified fatty acids
PG: plasma glucose
T2DM: type 2 diabetes mellitus
TEE: total energy expenditure
TG: triglyceride
